# An atypical erectile dysfunction patient with infertility treated with penile prosthesis implantation and testicular epididymal sperm aspiration (TESA)-intracytoplasmic sperm injection (ICSI): A case report

**DOI:** 10.1097/MD.0000000000034023

**Published:** 2023-06-23

**Authors:** Mengyuan Lin, Honghua Wang, Yan Wang, Shi-Wen Jiang

**Affiliations:** a Center of Reproductive Medicine, Women’s Hospital of Jiangnan University, Wuxi Jiangsu, China; b Center of Reproductive Medicine, State Key Laboratory of Reproductive Medicine, Research Institute for Reproductive Health and Genetic Diseases, Women’s Hospital of Jiangnan University, Wuxi Jiangsu, China.

**Keywords:** atypical erectile dysfunction, infertility, penile prosthesis implantation, testicular epididymal sperm aspiration

## Abstract

Erectile dysfunction (ED) is common in middle-aged and elderly men, affecting more than 100 million males worldwide. Most ED cases can be attributed to organic and/or psychological factors. Here we report an atypical ED case with no clear manifestation fitting the diagnosis for recognized types of ED.

**Patient concerns:**

The 35-year-old male is unable to have normal erection since puberty, and unable to complete intercourse with his wife. He had no history of trauma, surgery or psychiatric/psychological disease. The patient has a normal male karyotype. There is no significant finding in physical examination, nocturnal penile tumescence test, and ultrasound measurement of penis vascular functions. The serum levels of major hormones are all in normal ranges.

**Diagnoses:**

Atypical ED, psychogenic ED not excluded; infertility.

**Interventions:**

Oral phosphodiesterase inhibitors Tadalafil (20 mg, BIW) or Sildenafil (50 mg, BIW) had no effect in this patient. Penile prosthesis implantation helped the patient to acquire normal sexual life, but did solve the ejaculation failure and infertility. Motile sperms were obtained by testicular epididymal sperm aspiration under the guidance of ultrasound, and intracytoplasmic sperm injection was performed with occytes retrieved from his wife.

**Outcomes:**

The patient sexual life was significantly improved after penile prosthesis implantation; the patient wife is currently in the first trimester of pregnancy as the result of in vitro fertilization.

**Conclusions:**

The no response to phosphodiesterase type 5 inhibitors (PDE5) treatment may suggest an impediment of PDE5-related pharmacological pathways or the presence of defect/injury in the neural system. This special case raises a question if some patients with persistent ED may have similar manifestations and can be treated with the same procedures.

## 1. Introduction

Erectile dysfunction (ED) is defined by the Fourth International Consultation on Sexual Medicine as the consistent or recurrent inability to attain and/or maintain penile erection sufficient to accomplish intercourse.^[1]^ With more than 100 million patients worldwide, ED often occurs in men older than 40 years, and the incidence increases with age.^[2]^ By the age of 70, the incidence rate is higher than 50%. In the past decades, ED incidence rate is increasing in young men, possibly due to the changes of life style and accumulated stress from life and work.^[3,4]^ ED is considered an important factor for male infertility. Based on a study covering 7273 male infertility cases, WHO reported that ED accounts for 2.3% instances of unsuccessful fatherhood.^[5]^

ED can be attributed to various pathological causes including those related to psychogenic, organic or mixed factors. Organic ED can be further stratified into neurogenic, vasculogenic, endocrinological, and drug-induced subtypes. Psychogenic ED represents the most, nearly 60%, of ED cases,^[6]^ and depression-induced ED is well recognized. Clinically, psycho-sexual counseling, couple therapy and lifestyle modification are recommended firstly for patients without obvious organic defects. Drug treatment with phosphodiesterase type 5 inhibitors (PDE5) is often effective for psychogenic ED patients as well as some organic ED cases.^[7]^ If failed, intraurethral injection, vacuum constrictive device, or penile prosthesis are suggested for treatment.^[8]^ Recently, we encountered a rare case in the infertility clinic. The patient chief complaints and the physical/laboratory evidences support a diagnosis of ED, but did not fit either organic or psychogenic ED classification, and the patient did not respond to PDE5 therapy. This case special manifestations and treatment may deserve attention of physicians, especially those in the infertility clinic.

## 2. Case report

The patient is a 35-year-old male unable to erect since puberty. The failure of sexual life and infertility prompt the couple to our clinic. He has a history of nocturnal emission, but cannot complete intercourse and ejaculation. The patient has no medical history of trauma, surgery, diabetes, neurological or psychiatric/psychological diseases.

Physical examination shows a generally healthy male with well-developed penis and testis, and normal pubic hair growth and distribution. The foreskin is retractable and the penis head can be exposed. There is no rash or tissue injury around the penis and scrotum. The penis measures 7 cm in length and 2.5 cm in circumference, all in normal ranges.

Nocturnal penile tumescence test shows that in the sleep time of 8 to 9 hours, there are 3 to 6 erectile events with an average duration of 10 to 15 minutes. When erecting, the circumference of the penis increases by more than 2 cm, and the time for the hardness of the penis tip exceeding 70% lasts more than 10 minutes. Penile ultrasound: when the penis is flaccid, the diameter of the left cavernous artery of penis measures 0.05 cm, and the diameter of the right cavernous artery of penis measures 0.04 cm. After injection of prostaglandin E, the cavernous artery of the penis reaches: left PSV (peak systolic velocity), 40.45 cm/s; right PSV, 39.2 cm/s. The end diastolic velocity is 6.04 cm/s, and the RI (resistance index) is 0.88. All the results fall in normal ranges.

**Figure 1.**
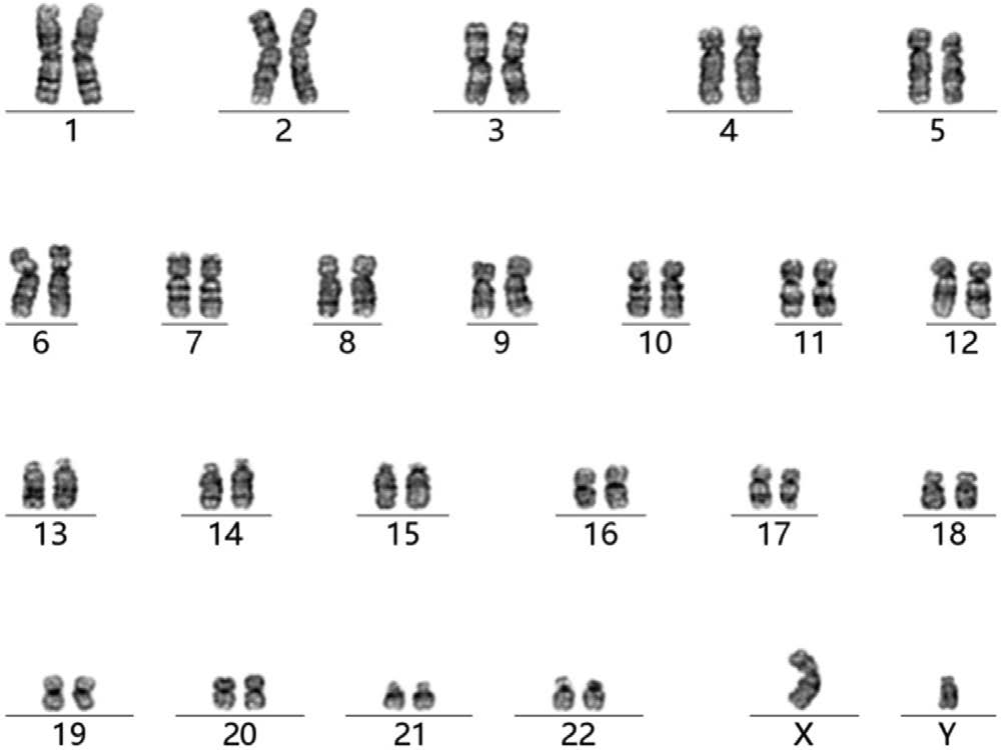
Results of karyotyping. Peripheral blood sample was obtained from the patient. White cell culture, colchicine treatment and G-banding was performed following routine procedures. Chromosomal analysis showed a 46, XY normal male karyotype, with no major defects detected.

Results of laboratory examination. The serum hormone levels are: follicle stimulating hormone, 3.45 mIU/mL; luteinizing hormone, 2.45 mIU/mL; estradiol, 24.7 pg/mL; prolactin 7.74 ng/mL; testosterone, 3.52 ng/mL; The test results are all in normal ranges. Chromosome examination (Fig. 1) shows a normal male karyotype without major chromosomal defect.

Diagnosis. Atypical ED, psychogenic ED not excluded; infertility.

**Figure 2.**
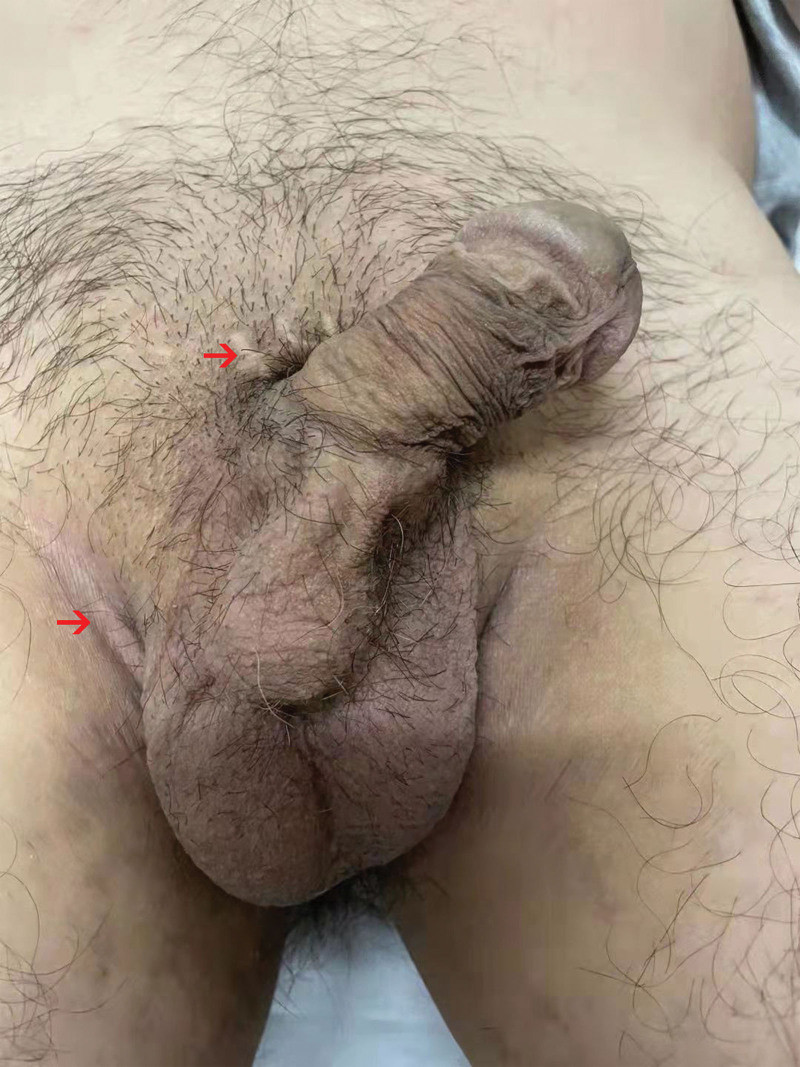
The patient received penile prosthesis implantation. The post-surgical scars are marked by red arrows.

**Figure 3.**
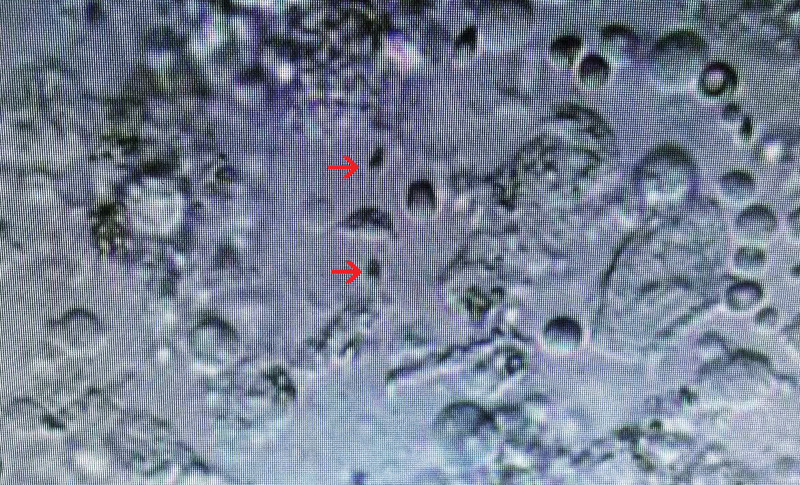
Testicular epididymal sperm aspiration was performed under the guidance of ultrasound. Motile sperms (indicated by red arrows) were obtained for intracytoplasmic sperm injection.

Treatment process. Oral phosphodiesterase inhibitors Tadalafil (20 mg, BIW) or Sildenafil (50 mg, BIW) had no effect. Penile prosthesis implantation was performed (Fig. 2). After operation, the patient acquired normal sexual life, but still could not discharge semen, and infertility persisted. We performed testicular epididymal sperm aspiration under the guidance of ultrasound, and obtained motile sperms (Fig. 3). The motile sperms were used for intracytoplasmic sperm injection. After implantation of in vitro fertilized oocytes, the patient wife is currently in the first trimester of pregnancy

## 3. Discussion

Multiple types of chromosomal abnormality can affect sex development and/or lead to sexual dysfunction such as ED.^[9–11]^ In addition, several ED-related genes have been uncovered, for example, the dysregulation of Myocardin gene expression has been found to be an important pathological mechanism for diabetic ED.^[12,13]^ Although this patient presents a normal male 46, XY karyotype, genetic mutation and aberrant gene expression cannot be excluded. Physical and ultrasonic examinations did not find organic defect or disease. The spontaneous erection at night and the positive response to penile injection also indicated that the patient does not belong to organic ED. Laboratory findings of normal serum levels of estradiol and testosterone helped to largely exclude the existence of major endocrine disorder.

From physiological point of view, erection is essentially a complex mussel-vascular function supported by cooperative actions of sycho-nuro-endocrine factors. Abnormality in any of these factors could cause ED. It is estimated that psychological factors account for 60% of ED cases.^[2]^ The diagnostic criteria for psychogenic ED^[14]^ are: The existence of psychiatric/psychological disease lasting for 6 months or longer; There is no evidence of organic defects or diseases. The penis hardness monitoring (nocturnal penile tumescence test) observes at least one effective erection (the penis head hardness ≥60%, and the erection duration ≥10 minute) for 3 consecutive nights. It is noteworthy that some investigators emphasize that psychogenic ED is a positive diagnosis that should not be used when the etiology of the disorder is uncertain or unknown.^[15]^ This patient is a hotel receptionist with college education, without history of psychiatric/psychological diseases. The patient has normal cognitive ability, appears to be optimistic and open-minded, and maintains a healthy life style. No sign of communication problems was observed in his interaction with doctors. The couple has intimate relationship, and both sides have desire and attempts for normal sexual life. All considered, the patient manifestations do not conform to the diagnostic criteria of organic or psychogenic ED.

Most ED patients have experience of effective erection, and their dysfunction can be traced to obvious age or sycho-physiogical factor(s). Moreover, 70% of ED patents can improve their erectile function by oral PED5 therapy.^[7]^ This patient never had an effective erection, and did not respond to PDE5 treatment, which may suggest the impediment of PDE5-related pharmacological pathways or the presence of defect or injury in the neural system. Nevertheless, psychological factor could not be excluded. The byphasic treatments achieved satisfactory outcomes. The penile prosthesis implantation helped the patient to improve his sexual life, and testicular epididymal sperm aspiration-intracy toplasmic sperm injection effectively addressed the reproductive needs. The diagnosis dilemma and treatment of such cases has not been documented in literature. This special case raises a question if some patients with persistent ED may have similar manifestations and can be treated with the same procedures.

## Author contributions

**Data curation:** Mengyuan Lin, Honghua Wang, Yan Wang.

**Funding acquisition:** Shi-Wen Jiang.

**Investigation:** Mengyuan Lin.

**Methodology:** Mengyuan Lin, Shi-Wen Jiang.

**Writing – original draft:** Mengyuan Lin, Shi-Wen Jiang.

**Writing – review & editing:** Mengyuan Lin, Honghua Wang, Shi-Wen Jiang.
